# Enrichment technique to allow early detection and monitor emergence of *KRAS* mutation in response to treatment

**DOI:** 10.1038/s41598-019-47700-9

**Published:** 2019-08-05

**Authors:** Yoshiyasu Kitagawa, Kazuhiro Okumura, Takayoshi Watanabe, Kei Tsukamoto, Shiro Kitano, Rino Nankinzan, Takuto Suzuki, Taro Hara, Hiroaki Soda, Tadamichi Denda, Taketo Yamaguchi, Hiroki Nagase

**Affiliations:** 1 Endoscopy Division, Chiba Cancer Center, Chiba, Japan; 2 Division of Oncogenomics, Chiba Cancer Center, Chiba, Japan; 3 Division of Cancer Genetics, Chiba Cancer Center, Chiba, Japan; 4 Technical Research Institute, TOPPAN PRINTING CO., LTD, Saitama, Japan; 5 Joint Research Laboratory (TOPPAN) for Advanced Cell Regulatory Chemistry, Graduate School of Engineering, Osaka University, Osaka, Japan; 6 Hara Clinic, Chiba, Japan; 7 Division of Gastroenterological Surgery, Chiba Cancer Center, Chiba, Japan; 8 Division of Gastroenterology, Chiba Cancer Center, Chiba, Japan

**Keywords:** Gene expression profiling, Diagnostic markers, Colon cancer, Cancer screening

## Abstract

Sensitivity of cell-free circulating tumour DNA (ctDNA) assays is often hampered by the limited quantity of intact mutant nucleotide fragments. To overcome the issue of substrate limitation in clinical applications, we developed an enrichment method utilizing pyrrole-imidazole (PI) polyamides and their ability to bind the minor groove of B-DNA. We present here a proof-of-concept experiment to enrich specific mutant *KRAS* alleles with biotinylated PI polyamides. We investigated the clinical feasibility of incorporating PI polyamides to detect *KRAS* mutations in ctDNA from 40 colorectal cancer (CRC) patients, of whom 17 carried mutations in *KRAS*. After enriching ctDNA with those polyamides, we used digital PCR to detect several common *KRAS* codon 12 mutations. Enrichment by biotinylated PI polyamides improved the sensitivity of ctDNA analysis (88.9% vs. 11.1%, *P* < 0.01) in 9 non-metastatic mutation-positive patients. We observed no differences in performance for the 8 metastatic subjects (100% vs. 75%, *P* = 0.47). In the remaining 23/40 patients with wild type *KRAS* codon 12, no mutant alleles were detected with or without polyamide-facilitated enrichment. Enriching B-form of ctDNA with PI polyamides significantly improved the assay sensitivity in detecting *KRAS* mutations in non-metastatic CRC patient samples.

## Introduction

Mutations in ras genes, particularly H-ras, N-ras, and K-ras, are commonly identified in many types of tumors and have been implicated in the development of human cancer^1,2^. Of these genes, mutations in *KRAS* are much more frequent than *NRAS* or *HRAS*^3^, with a frequency of 90% in pancreatic cancer^4^, 35% in lung cancer^5^, and 30–60% in colon cancer^6,7^. Thus, *KRAS* mutations are predicted to be promising biomarkers for the early diagnosis of primary tumors.

Currently, histological examination of tumor tissues is the gold standard for diagnosing the presence of mutations in *KRAS*. However, examining tissue samples has some disadvantages, as tumors and metastases are not always accessible for biopsy, collection of biopsies often requires invasive procedures, and intratumoral heterogeneity is not well understood.

An alternative approach to overcome these issues is the analysis of cell-free circulating tumor DNA (ctDNA). Several groups are currently developing methods to detect somatic mutations with the aim of discovering markedly higher cell-free DNA (cfDNA) concentrations in the blood of patients with cancer, in particular, *KRAS* from plasma and serum^8–11^. New techniques, such as digital polymerase chain reaction (dPCR)^12,13^, as well as beads, emulsions, amplification, and magnetics (known as BEAMing)^14,15^, allow highly sensitive detection of cancer-specific DNA in the blood. In patients with late-stage cancer, measurement of ctDNA can be used as a liquid biopsy to predict response to targeted therapies^11,12,15^.

Use of ctDNA measurement also raises the possibility of screening and diagnosing before cancers becomes clinically detectable^12,16^. However, the diagnostic sensitivity of ctDNA analysis for cancer detection is often too low due to very low levels of ctDNA and detection of background of nontumor-derived cfDNA^13^. Therefore, patients can only be diagnosed at advanced stages with high tumor burden and high ctDNA concentrations.

Due to the difficulties in detecting cancer-specific mutations at the early disease stage, we developed a novel approach to specifically enrich mutant *KRAS* alleles in ctDNA using biotinylated pyrrole–imidazole (PI) polyamides. Hairpin pyrrole (Py)–imidazole (Im) polyamides bind with high affinity to the minor groove of specific sequences of the Watson–Crick B-form DNA; Py moieties preferentially bind T, A, and C bases, but not G, whereas Im is a G-reader^17^. Therefore, we synthesized biotinylated PI polyamides to target common *KRAS* codon 12 mutations to enhance the detection of *KRAS* mutations in ctDNA. The results from the dPCR analysis were compared before and after the enrichment assay and demonstrated an increased sensitivity using this approach to detect *KRAS* mutations in patients with colorectal cancer (CRC).

## Results

### Biotinylated PI polyamides target KRAS codon 12 mutants

Biotinylated PI polyamide (KRAS5) was designed to recognize *KRAS* G12V/G12D mutations. Surface plasmon resonance (SPR) analysis was conducted to evaluate the binding affinities of KRAS5 to the target DNAs, *KRAS* G12D (GAT), *KRAS* G12V (GTT), and *KRAS* wild type (GGT) sequence, as described previously (Supplementary Fig. S1 and Table 1)^18^. *KRAS* mutations (G12D and G12V) showed substantial SPR responses, while wild type sequence did not show a detectable signal, suggesting that KRAS5 was unable to bind to the wild type sequence. Dissociation equilibrium constants (K_D_ = *kd*/*k*a, where *kd* and *k*a are the dissociation and association rate constants, respectively) between the free and bound states of KRAS5 were determined by fitting the SPR results as an exponential decay model (Supplementary Fig. S1)^19^. G12D (3.04 × 10^−7^ M) and G12V (2.45 × 10^−7^ M) were two orders of magnitude lower than the wild type (167 × 10^−7^ M, Table 1) in *KD*, indicating that KRAS5 had a lower affinity for the wild type sequence (Table 1).

**Table 1:** Binding affinities of KRAS5 with DNAs of *KRAS* mutations (G12D and G12V) or *KRAS* wild type (WT).

Status	Sequecne	K_D_[10^−7^ M]^a^	*k*_*a*_[10^3^ M^−1^ S^−1^]^b^	*k*_*d*_[10^−3^ S^−1^]^c^
G12D	GAT	3.04	79.3	2.41
G12V	GTT	2.45	105	2.58
WT	GGT	167	1.13	1.88

^a^Dissociation Constant. ^b^Association rate constant. ^c^ Dissociation rate constant.

### Fold enrichment in the titration study

Each plasmid DNA (G12V, G12D, G12S, and G12C) was spiked into 50 ng of whole blood DNA, and titration samples (fraction of mutant alleles: 10%, 1%, and 0.1%) were prepared. An enrichment assay was then performed using these samples. The fold enrichment was calculated from the ddPCR results, and defined as the fractional abundance of the assay divided by that of the titration samples.

**Figure 1:**
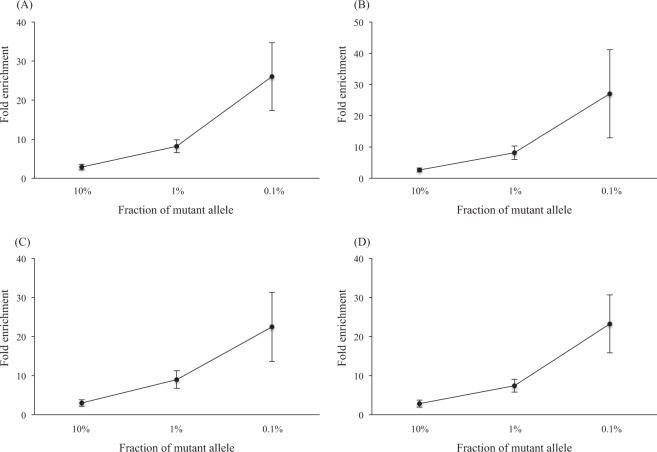
Relationship between fold of enrichment assay and fraction of mutant alleles. (**A**) Fold enrichment of KRAS5 for G12V alleles. (**B**) Fold enrichment of KRAS5 for G12D alleles. (**C**) Fold enrichment of KRAS4 for G12S alleles. (**D**) Fold enrichment of KRAS4 for G12C alleles.

First, a multiplex analysis was performed for each plasmid sample. For the G12V alleles, the mean (±SD) fold of the enrichment assay in the 10%, 1%, and 0.1% mutant fractions were 2.85 ± 0.74, 8.17 ± 1.66, and 25.99 ± 8.70, respectively. For the G12D alleles, the mean folds in the 10%, 1%, and 0.1% mutant fraction were 2.63 ± 0.60, 8.15 ± 2.15, and 26.98 ± 14.08, respectively. For the G12S alleles, the mean folds in the 10%, 1%, and 0.1% mutant fraction were 2.98 ± 0.81, 9.02 ± 2.28, and 22.45 ± 8.84, respectively. Finally, for the G12C alleles, the mean folds in the 10%, 1%, and 0.1% mutant fraction were 2.85 ± 0.90, 7.42 ± 1.61, and 23.24 ± 7.40, respectively. For each *KRAS* mutation, the folds were significantly higher in the 1% mutant fraction compared with the 10% mutant fraction (*P* < 0.05) and were also higher in the 0.1% mutant fraction compared with 1% mutant fraction (*P* < 0.05) (Fig. 1). When we performed additional experiments, a similar tendency was obtained in the 0.01% mutant fraction (Supplementary Table 1).

To confirm the results obtained using multiplex ddPCR, we conducted additional analyses with a duplex ddPCR approach, in which only two molecular targets were detected in each experiment (i.e., wild type and a given mutant sequence)^12^. For the G12V alleles, the mean (±SD) folds of the enrichment assay in the 10%, 1%, and 0.1% mutant fraction were 2.07 ± 0.22, 9.66 ± 2.26, and 42.24 ± 6.87, respectively. For the G12D alleles, the mean folds in the 10%, 1%, and 0.1% mutant fraction were 1.99 ± 0.26, 8.65 ± 1.13, and 36.54 ± 6.88, respectively. For each *KRAS* mutation, the mean folds were significantly higher in the 1% mutant fraction compared with the 10% mutant fraction (*P* < 0.05) and were also higher in the 0.1% mutant fraction compared with the 1% mutant fraction (*P* < 0.05) (Supplementary Fig. S2).

### Patients’ characteristics

Table 2 shows a summary of the patients’ characteristics. The median patient age was 64 (range 39–82) years and there was a preponderance of males (n = 27, 67.5%). Primary tumors were located in the right-sided colon in 12 (30.0%) patients, and the left-sided colon in 28 (70.0%) patients. Two (5.0%), four (10.0%), 11 (27.5%), and 23 (57.5%) patients had stage I, II, III, and IV cancer, respectively. The median carcinoembryonic antigen (CEA) level was 23.5 ng/mL (range, 1.7–7317.9 ng/mL). Tumor characterization revealed that 17 patient tumors were positive for *KRAS* mutations, of which six (35.3%) were G12V mutations and 11 (64.7%) were G12D.

**Table 2:** Patients’ characteristics.

	N = 40
Age, median (range), years	64 (39–82)
Sex (male:female)	27:13
Location (right-sided: left-sided)	12:28
**Stage of disease (UICC)**	
I	2
II	4
III	11
IV	23
CEA (ng/mL)	23.5 (1.7–7317.9)
***KRAS*** **, primary tumour**	
wild-type	23
mutants (G12V:G12D)	17 (6:11)

CEA, carcinoembryonic antigen.

### *KRAS* mutation detection in ctDNA

The median serum DNA concentration in CRC patients was 107.5 ng/mL (range, 0.0–3660.0 ng/mL), and the median DNA concentration in metastatic CRC patients was significantly higher than that of non-metastatic CRC patients (0.0 ng/mL vs. 359.0 ng/mL, *P* < 0.01).

First, dPCR analysis was performed in 40 ctDNA samples prior to the enrichment assay. The same samples were then analyzed by dPCR using biotinylated PI polyamides (KRAS5). Then, the dPCR analysis results were compared before and after the enrichment assay.

Supplementary Table 2 summarizes the findings of the dPCR analysis. *KRAS* mutations in ctDNA were detected in nine of the 40 (17.5%) patients before the enrichment assay and 16 of the 40 (40.0%) patients after the enrichment assay. Supplementary Fig. S3 shows representative cases (cases 1–9).

Table 3 shows concordance of the data from the tumor-tissue analysis and ctDNA analysis. The diagnostic accuracy of the ctDNA analysis after enrichment assay was significantly greater than that before the enrichment assay (97.5% vs. 75.0%, *P* < 0.01). After the enrichment assay, ctDNA analysis had greater diagnostic sensitivity compared with that before the enrichment assay (94.1% vs. 41.2%, *P* < 0.01). While case 4 did not reach the cutoff value of 1.0 copy, there was a tendency toward an increase in mutant DNA after enrichment (Supplementary Fig. S3). The diagnostic specificities before and after the enrichment assay were not significantly different (100% vs. 100%, *P* = 0.49).

**Table 3:** Concordance between tumor-tissue analysis and ctDNA analysis.

	Before enrichment assay	Tumor-tissue analysis				
		Mutant	WT	Accuracy	Sensitivity	Specificity
	After enrichment assay	Mutant	WT	Accuracy	Sensitivity	Specificity
ctDNA analysis	Mutant	7	0	75.0%	41.2%	100%
	WT	10	23			
	Total	17	23			
ctDNA analysis	Mutant	16	0	97.5%^a^	94.1%^a^	100%^b^
	WT	1	23			
	Total	17	23			

^a^*P* < 0.05 vs. before enrichment assay.

^b^No significant difference compared with before enrichment assay.

**Figure 2:**
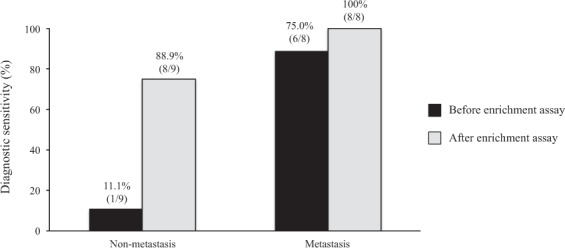
Diagnostic sensitivities, in terms of pathological stages.

Figure 2 shows the diagnostic sensitivities in terms of pathological stages. Among the nine patients with non-metastatic CRC bearing tumor-associated *KRAS* mutations, the diagnostic sensitivity after enrichment assay was significantly higher than that before the enrichment assay (88.9% vs. 11.1%, *P* < 0.01). Whereas, among the eight patients with metastatic CRC bearing tumor-associated *KRAS* mutations, the diagnostic sensitivities before and after the enrichment assay were not significantly different (100% vs. 75.0%, *P* = 0.47).

## Discussion

We developed a novel PI polyamide-based enrichment approach to target common *KRAS* codon 12 mutations (G12V, G12D, G12S, and G12C). In the titration study, greater enrichment effects were achieved in samples containing a lower fraction of mutant *KRAS* alleles. Our approach significantly improved the identification of *KRAS* mutations in ctDNA, particularly from patients with non-metastatic CRC.

Hairpin Py–Im polyamides can be designed for specific 9-base pair recognition of the genome following Dervan’s recognition rule, as seen in natural minor groove binders, which undergo hydrogen bonding to DNA^17,20^. We demonstrated previously direct targeting of *KRAS* mutant DNA using alkylating polyamide conjugates that selectively recognize oncogenic codon 12 *KRAS* mutations^17^. In the present study, we designed biotinylated PI polyamides to enrich mutant *KRAS* alleles in the presence of an excess of wild type alleles.

ctDNA analysis, based on the “liquid biopsy” concept, was recently reported to represent a promising biomarker in patients with various types of cancer^21,22^. In metastatic CRC, identification and quantification of ctDNA can be used to evaluate response to treatment and assess the real-time development of resistance^11,12,15,23^. The reported diagnostic sensitivity ranged from 77.8% to 97.2%^11,12,15^. Before the enrichment assay, our results showed a sensitivity of 75.0%, which was similar to but not as high as previous reports. These results may have been due to use of a small sample volume of 500 μL. Despite this limitation, we found a very high sensitivity (100%) after the enrichment assay. Therefore, our enrichment assay could be used in a cohort study with limited sample volumes, such as residual serum samples after blood chemistry analysis.

Regarding early diagnosis of CRC, there are few reports on the sensitivity of ctDNA analysis to detect *KRAS* mutations. A previous report showed that mutant serum *KRAS2* was detectable even in cases of small colonic polys^24^. However, this study did not use quantitative analysis and did not have matched tumor and serum samples for all cases. Another recent study on non-metastatic CRC was able to detect 5.6% of tumor mutations by dPCR^25^, which is not satisfactory as an early diagnostic biomarker. Thus, further improvement of techniques is required to define patients with early-stage CRC. The present study on non-metastatic CRC demonstrated that ctDNA analysis achieved an extremely high degree of diagnostic sensitivity (88.9%) following the enrichment assay. This may represent the best opportunity to cure CRC. One possible reason for the significant improvement in patients with non-metastatic CRC is that PI polyamides increased the selectivity when the fraction of mutant *KRAS* alleles decreased. This improvement is not likely artificial, since ctDNA analysis after enrichment assay achieved 100% specificity.

In CRC patients, early detection of recurrence after surgery during follow-up is associated with improved survival^26–29^. Computed tomography imaging improves the detection of recurrence, but is associated with radiation exposure. Furthermore, it can only detect residual disease when a sufficient tumor bulk is present^29,30^. While CEA is currently the standard biomarker used, it has limited sensitivity and specificity^30,31^. Thus, further improvement of strategies is required to detect minimal residual disease. Postoperative ctDNA detection provides direct evidence of remaining cancer cells and can identify patients at high risk of relapse^28,32^. In the present study, among patients with non-metastatic CRC bearing tumor-associated *KRAS* mutations, we were able to detect *KRAS* mutations in ctDNA after enrichment assay of CEA-negative patients. While our approach is not a perfect indicator, as biotinylated PI polyamides only targeted four *KRAS* mutations, its sensitivity is attractive. If ctDNA analysis after enrichment assay can reliably detect relapse, this would enable patients eligible for curative surgical resection or earlier implementation of systematic therapies to be identified. In addition, the potential benefit of this approach may have clinical implication in monitoring resistance to treatment and emergence of new *KRAS* mutations. Further study involving the longitudinal monitoring of patients is required to validate whether our enrichment technique allows early detection and monitor emergence of *KRAS* mutation after treatment.

Pancreatic cancer exhibits a high frequency (75–95%) of *KRAS* mutations^4,33^. Furthermore, in pancreatic cancer, the most frequent *KRAS* point mutations are located in two consecutive nucleotides in codon 12^4^. Therefore, *KRAS* codon 12 mutations in ctDNA represent an important potential biomarker of pancreatic cancer, which is fatal disease that is often diagnosed at advanced stages due to a lack of suitable techniques for early detection and diagnosis^22,34^. However, the role of *KRAS* mutations in the early diagnosis of pancreatic carcinoma remains controversial^35^, because the diagnostic sensitivity of *KRAS* analysis is low (35–38%)^13,36^. However, we believe that our enrichment assay could be advantageous in improving the diagnosis of *KRAS*-mutated ctDNA and could be also used for the early detection of pancreatic cancer.

This study had several limitations as it was a single-center, retrospective, small study that used residual serum samples. In a previous study, the detection rates of *KRAS* mutations in serum and plasma DNA were almost equal, and both serum and plasma DNA could be used to detect prognostic biomarkers for pancreatic cancer^36^. However, the use of plasma is recommended for the analysis of ctDNA due to lower concentrations of background wild type DNA^37^. Further studies involving larger numbers of patients and using plasma samples is required to validate the utility of the enrichment of mutant *KRAS* alleles. Nonetheless, our approach clearly shows sufficiently improved detection of *KRAS* mutations in ctDNA from patients with non-metastatic CRC.

In conclusion, enrichment of mutant *KRAS* alleles using biotinylated PI polyamides represents a feasible and effective method to identify *KRAS* mutations in ctDNA. Our results support the requirement for further evaluation of this newly developed approach to detect early diagnostic biomarkers for the management of CRC patients.

## Materials and Methods

### Synthesis of biotinylated PI polyamides

**Figure 3:**
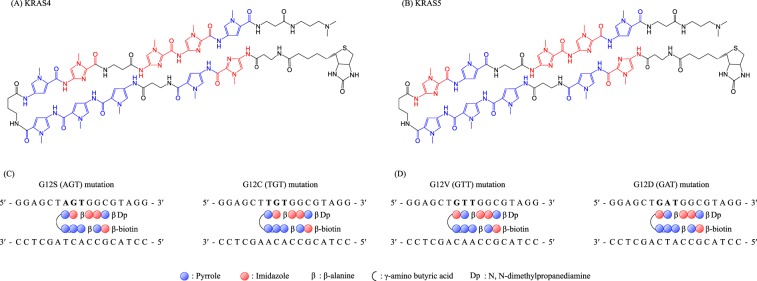
Structure of biotinylated pyrrole–imidazole polyamides. (**A**) Chemical structure of the PI polyamide, KRAS4. (**B**) Chemical structure of the PI polyamide, KRAS5. (**C**) KRAS4 targets G12S/G12C mutations. (**D**) KRAS5 targets G12V/G12D mutations.

Figure 3 shows the structures of the biotinylated PI polyamides (KRAS4 and KRAS5). KRAS4 and KRAS5 were designed to recognize *KRAS* G12S/G12C and G12V/G12D mutations, respectively (Fig. 3). Biotinylated PI polyamides were synthesized using a stepwise reaction according to a previously described Fmoc solid phase protocol^18,38–40^. Synthesis was performed using a Peptide Synthesizer (PSSM-8, Shimadzu Industry) with a computer-assisted operation system on a 10 μmol scale (19.6 mg of Fmoc–β-alanine Wang resin). Following synthesis, *N*,*N*-dimethylpropanediamine (Dp) was mixed with the resin then heated to 65 °C for 2 h to detach the PI polyamides from the resin. Purification of PI polyamides was performed using a high-performance liquid chromatography (HPLC) LC-20 (SHIMAZU Industry), using a 10 mm × 150 mm Phenomenex Gemini-NX3u 5-ODS-H reverse-phase column in 0.1% acetic acid in water with acetonitrile as the eluent, a flow rate of 10 mL/min, and a linear gradient from 0% to 66.7% acetonitrile over 20 min, with detection at 310 nm. Collected fractions were analyzed by liquid chromatography mass spectroscopy. After purification, N-terminal biotinylation was performed to produce KRAS4 and KRAS5 by incubating the cleavage product in the presence of biotin, PyBOP, and DIEA for 2 h. After solvent evaporation, the reaction product was characterized and purified by liquid chromatography (Shimadzu, Prominence HPLC) using a linear gradient of acetic acid (0.1%) in acetonitrile and water (30–75%, linear over 30 min). Peaks were visualized at 310 nm for compound characterization.

### Surface plasmon resonance assay

All SPR experiments were performed on a Biacore X100 (GE Healthcare) at 25 °C as described previously^18^. Biotinylated hairpin KRAS G12D mutated nucleotide (5′biotin- TACATCTGATGGCGTATTAAGTTTTCTTAATACGCCATCAGATGTA-3′, Hokkaido System Science), KRAS G12V mutated nucleotide (5′biotin- TACATCTGTTGGCGTATTAAGTTTTCTTAATACGCCAACAGATGTA-3′, Hokkaido System Science) and KRAS wild nucleotide (5′biotin- TACATCTGGTGGCGTATTAAGTTTTCTTAATACGCCACCAGATGTA-3′, Hokkaido System Science) were immobilized on a streptavidin coated SA sensor chip at a flow rate of 10 μL/min to obtain the required immobilization level (up to approximately 1000 resonance units (RU)). Samples were dissolved in HBS-EP buffer (10 mM 4-(2-hydroxyethyl)- 1-piperazineethanesulfonic acid (HEPES), 150 mM NaCl, 3 mM ethylenediamine tetraacetic acid (EDTA), and 0.005% surfactant P20) with 0.1% DMSO at 25 °C, pH 7.4 for the experiment.

### Enrichment protocol using biotinylated PI polyamides

**Figure 4:**
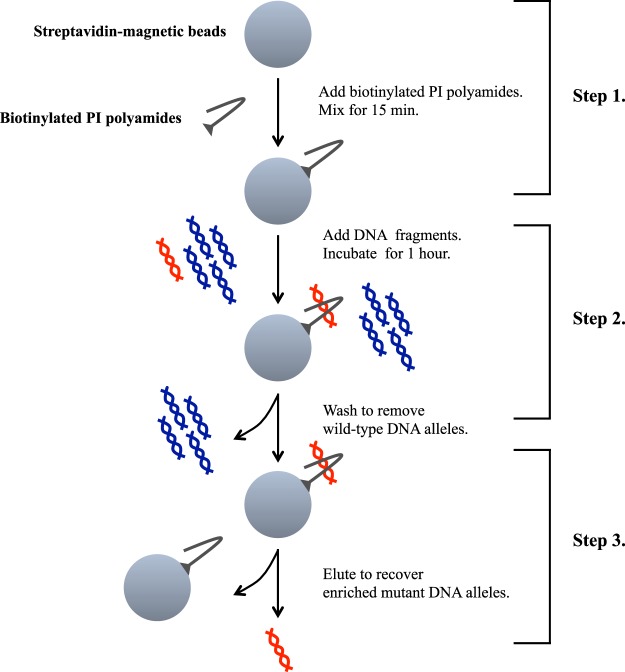
Schematic representation of the enrichment assay using biotinylated pyrrole–imidazole polyamides.

Figure 4 shows a schematic representation of the enrichment assay and outlines its three distinct steps^1^. Preparation of the magnetic beads was performed as follows. After removing the suspension solution from streptavidin magnetic beads (Magnosphere MS300/Streptavidin, JSR Corporation, Tokyo, Japan), the beads were washed with buffer (10 mM Tris–HCl, pH 7.4); 0.5 mM EDTA; 1 M NaCl; and 0.1% Tween20) and then resuspended in the same buffer. The resuspended streptavidin magnetic beads were mixed with biotinylated PI polyamides and centrifuged at 1300 rpm for 15 min. After removing the buffer from beads and biotinylated PI polyamides, the beads were washed with buffer and resuspended in water^2^. Beads and biotinylated PI polyamides were then incubated with DNA fragments while being centrifuged at 1300 rpm for 1 h. After removing the supernatant solution and wild type DNA alleles, mutant DNA alleles with beads and biotinylated PI polyamides were washed with water^3^. Then, enriched mutant DNA alleles were resuspended in Tris–EDTA buffer and DNA was eluted from the beads and biotinylated PI polyamides after heating at 97 °C for 30 min.

### Patients

A total of 40 tumor samples and matched serum samples were obtained from 40 patients between October 2010 and November 2016 at the Chiba Cancer Center (Chiba, Japan), and were analyzed in the present study. The residual serum samples used in the study were frozen and stored immediately after routine blood chemistry analysis prior to surgical treatment. All the samples were histologically diagnosed as CRC and were obtained prior to therapy. None of the patients received preoperative chemotherapy or radiotherapy. As controls, 10 healthy individuals were recruited with no prior history of any cancer.

All the patients provided written informed consent for use of their serum and clinical data. The present study was reviewed and approved by the ethics committee of the Chiba Cancer Center, and the protocol was displayed on a notice board for inpatients and outpatients (number 1875). The study was carried out in accordance with the World Medical Association’s Declaration of Helsinki.

### Analysis of *KRAS* mutations in colorectal tumor tissue

Tissue samples were collected by biopsy or surgical resection. DNA was purified from formalin-fixed, paraffin-embedded specimens using a QIAamp DNA Mini kit (Qiagen, Valencia, CA, USA) according to the manufacture’s protocol^41^. Analysis of *KRAS* mutation in primary tumors was performed using an amplification refractory mutation system (ARMS)-Scorpion or Luminex assays as described previously^42,43^.

### Blood collection and cfDNA extraction

Blood samples were collected from patients into tubes containing EDTA at the first visit. The samples were centrifuged at 3000 × *g* at 4 °C to separate serum from peripheral blood cells and, following blood chemistry analysis, samples were stored at −80 °C until analyzed further. ctDNA was extracted from 500 μL of serum using the Maxwell RSC ccfDNA Plasma Kit and an automated Maxwell RSC Instrument (Promega, Madison, Wisconsin, USA) according to the manufacturer’s protocol, as described previously^44^. Extracted DNA was quantified using a Qubit fluorometer (Thermo Fisher Scientific, Waltham, MA, USA).

### Droplet digital PCR

We used a QX200 droplet digital PCR (ddPCR) system (Bio-Rad, Hercules, CA, USA) to analyze the presence of mutations and wild type status in *KRAS*, as described previously^36,44^. ctDNA from CRC patients and plasmid DNA were amplified using the ddPCR *KRAS* Screening Multiplex Kit (Bio-Rad) capable of detecting the seven most common *KRAS* mutations. To confirm the results obtained using the multiplex ddPCR, an additional analysis was performed using a duplex ddPCR approach using the Prime-PCR ddPCR Mutation Detection Assay Kit (Bio-Rad) (G12V and G12D). A volume of 8 μL of DNA template was added to 10 μL of ddPCR Supermix for Probes (Bio-Rad) and 2 μL of the primer/probe mixture. Droplets were generated using the Automated Droplet Generator (Auto-DG, Bio-Rad), where the reaction mix was added together with Droplet Generation Oil for Probes (Bio-Rad). The emulsion was added to a thermal cycler, starting with enzyme activation of 10 min at 95 °C, followed by 40 cycles of 30 s at 94 °C and 1 min at 60 °C, and finishing with 10 min at 98 °C for enzyme deactivation. When the cycling was complete, the fluorescence signal was measured for each droplet. Droplets were analyzed using the QX200 Droplet Reader (Bio-Rad) for fluorescent measurement of FAM and HEX probes. Data from the fluorescence signals were analyzed using the Quanta software program, version 1.4.0 (Bio-Rad), in accordance with the manufacturer’s protocol, to determine the number of droplets positive for wild type *KRAS* and/or *KRAS* mutations. In the present study, the cutoff values for mutations were determined using serum cfDNA from 10 healthy individuals, and were set at 1.0 copy per reaction.

### Titration study using plasmid DNA and genomic DNA

A titration study was initially performed to evaluate the enrichment of targeted DNA alleles using plasmid DNA containing four *KRAS* mutations (G12V, G12D, G12S, and G12C) and wild type genomic DNA isolated from human whole blood. Plasmid DNAs were amplified by PCR and cloned into the plasmid, pCRH2.1 (Thermo Fisher Scientific, Waltham, MA, USA)^41^. The synthesized templates were verified by sequencing and used for the titration study (fraction of mutant alleles: 10%, 1%, and 0.1%).

### Statistical analysis

Means and standard deviations (SDs) of fold enrichments were calculated for the enrichment assay in the titration samples. Fold enrichment between titration samples was compared using a paired *t* test. For diagnostic performance, accuracy, sensitivity, and specificity, data are presented as percentages. The diagnostic performance of each modality was statistically analyzed by Fisher’s exact test. *P* < 0.05 for two-tailed tests was regarded as significant. SPSS software, version 17.0 (SPSS Inc, Chicago, IL, USA) was used to perform statistical analyses.

## Supplementary information

Supplementary information
