# Supplementary material for: Enrichment technique to allow early detection and monitor emergence of KRAS mutation in response to treatment
Source: Sci Rep. 2019 Aug 5;9:11346. doi: 10.1038/s41598-019-47700-9 (PMC6683117; doi:10.1038/s41598-019-47700-9)
Supplement: Supplementary file 1 — Supplementary information [file 41598_2019_47700_MOESM1_ESM.docx]

**Enrichment technique to allow early detection and monitor emergence of *KRAS* mutation in response to treatment**

Yoshiyasu Kitagawa^1,2^, Kazuhiro Okumura^2^; Takayoshi Watanabe^2^, Kei Tsukamoto^3^, Shiro Kitano^3^, Rino Nankinzan^1^, Takuto Suzuki^1^, Taro Hara^4^; Hiroaki Souda^5^, Tadamichi Denda^6^, Taketo Yamaguchi^6^, Hiroki Nagase^2^

**Corresponding author:** Hiroki Nagase, MD, PhD

Division of Cancer Genomics, Chiba Cancer Center, 666-2 Nitonacho, Chuo-ku, Chiba, Japan

Phone: 043-264-5431, Fax: 043-262-8680, Email: hnagase@chiba-cc.jp

^1^Endoscopy Division, Chiba Cancer Center, Chiba, Japan; ^2^Division of Cancer Genomics, Chiba Cancer Center, Chiba, Japan; ^3^TOPPAN PRINTING CO., LTD., Technical Research Institute, Saitama, Japan; ^4^Hara Clinic, Chiba, Japan; ^5^Division of Gastroenterological Surgery, Chiba Cancer Center, Chiba, Japan; ^6^Department of Gastroenterology, Chiba Cancer Center, Chiba, Japan

SUPPLEMENTARY INFORMATION

Supplementary Table. 1–2, with legends

Supplementary Fig. S1–S3, with legends

**SUPPLEMENTARY INFORMATION**

**SUPPLEMENTARY TABLES**

**Table S1** Additional experiments to evaluate the limit of enrichment assay using plasmid DNA.

| Status | G12D　* | G12C　* |
| --- | --- | --- |
| Number of experiments | 10 | 20 |
| Mean folds of enrichment assay | 397.0 | 909.1 |
| Maximum folds of enrichment assay | 769.1 | 10000.0 |

* 　Each plasmid DNA containing *KRAS* mutations (G12C and G12D) was mixed with wild-type DNA, and titration samples (fraction of mutant alleles: 0.01%) were prepared.

**　The fold enrichment was calculated from the ddPCR results, and defined as the fractional abundance of the assay divided by that of the titration samples.

**Table S2** Digital PCR analysis of ctDNA samples before and after enrichment assay.

|  | | | | | | | | **Before enrichment assay** | | **After enrichment assay** | |
| --- | --- | --- | --- | --- | --- | --- | --- | --- | --- | --- | --- |
| **Case** | **Sex** | **Age, years** | **Primary site** | **Stage** | **CEA,** | **Concentration,** | ***KRAS* status,** | **Mutant (Copies/μL)** | **FA (%)** | **Mutant (Copies/μL)** | **FA (%)** |
|  |  |  |  |  | **< 5.0 ng/mL** | **ng/mL of serum** | **primary** | **Wild (Copies/μL)** |  | **Wild (Copies/μL)** |  |
| 1 | M | 71 | Rectum | Ⅰ | 3.4 | 60 | G12D | n.d. | – | 13.9 | 81.8 |
|  |  |  |  |  |  |  |  | 1.6 |  | 3.1 |  |
| 2 | F | 82 | A-colon | Ⅰ | 2.5 | < 20 | G12V | n.d. | – | 5.9 | 79.7 |
|  |  |  |  |  |  |  |  | 1.5 |  | 1.5 |  |
| 3 | M | 73 | Rectum | Ⅱ | 10 | < 20 | G12V | 1.7 | 48.6 | 5.2 | 78.8 |
|  |  |  |  |  |  |  |  | 1.8 |  | 1.4 |  |
| 4 | M | 71 | Cecum | Ⅱ | 4.8 | < 20 | G12V | n.d. | – | n.d. | – |
|  |  |  |  |  |  |  |  | 1.2 |  | n.d. |  |
| 5 | M | 79 | A-colon | Ⅲ | 6.3 | < 20 | G12D | n.d. | – | 20.1 | 61.8 |
|  |  |  |  |  |  |  |  | 1.6 |  | 12.4 |  |
| 6 | M | 45 | Rectum | Ⅲ | 22.5 | 60 | G12D | n.d. | – | 8.6 | 70.5 |
|  |  |  |  |  |  |  |  | 5.5 |  | 3.6 |  |
| 7 | F | 72 | Cecum | Ⅲ | 40.2 | < 20 | G12D | n.d. | – | 4.1 | 75.9 |
|  |  |  |  |  |  |  |  | 4.5 |  | 1.3 |  |
| 8 | M | 49 | S-colon | Ⅲ | 12.5 | < 20 | G12D | n.d. | – | 3 | 63.8 |
|  |  |  |  |  |  |  |  | 1.2 |  | 1.7 |  |
| 9 | M | 65 | S-colon | Ⅲ | 18.5 | 122 | G12V | n.d. | – | 5.5 | 68.8 |
|  |  |  |  |  |  |  |  | 9.3 |  | 2.5 |  |
| 10 | F | 74 | Cecum | Ⅳ | 12.4 | 199 | G12D | n.d. | – | 1.9 | 37.3 |
|  |  |  |  |  |  |  |  | 5 |  | 3.2 |  |
| 11 | F | 77 | S-colon | Ⅳ | 101.5 | 120 | G12D | 1.9 | 21.1 | 3 | 40.5 |
|  |  |  |  |  |  |  |  | 7.1 |  | 4.4 |  |
| 12 | F | 49 | Rectum | Ⅳ | 24.5 | 343 | G12D | 9 | 22.6 | 7 | 42.4 |
|  |  |  |  |  |  |  |  | 30.9 |  | 9.5 |  |
| 13 | F | 68 | A-colon | Ⅳ | 329.5 | 231 | G12V | 7.1 | 26.3 | 5.1 | 34.7 |
|  |  |  |  |  |  |  |  | 19.9 |  | 9.6 |  |
| 14 | M | 61 | D-colon | Ⅳ | 203 | 1040 | G12D | 48 | 33.6 | 7 | 34.1 |
|  |  |  |  |  |  |  |  | 95 |  | 13.5 |  |
| 15 | F | 56 | S-colon | Ⅳ | 717 | 1690 | G12V | 7.9 | 0.6 | 3.9 | 1.5 |
|  |  |  |  |  |  |  |  | 1240 |  | 250 |  |
| 16 | M | 74 | A-colon | Ⅳ | 498.4 | 3660 | G12D | 42 | 38.4 | 12.5 | 43.1 |
|  |  |  |  |  |  |  |  | 67.4 |  | 16.5 |  |
| 17 | F | 47 | S-colon | Ⅳ | 10.9 | 100 | G12D | n.d. | – | 1.4 | 31.8 |
|  |  |  |  |  |  |  |  | 11 |  | 3.1 |  |
| 18 | M | 68 | S-colon | Ⅱ | 21.2 | < 20 | WT | n.d. | – | n.d. | – |
|  |  |  |  |  |  |  |  | 1 |  | n.d. |  |
| 19 | F | 65 | S-colon | Ⅱ | 10.6 | 60 | WT | n.d. | – | n.d. | – |
|  |  |  |  |  |  |  |  | 7.2 |  | 2.8 |  |
| 20 | M | 60 | Rectum | Ⅲ | 4.6 | < 20 | WT | n.d. | – | n.d. | – |
|  |  |  |  |  |  |  |  | 2.1 |  | n.d. |  |
| 21 | M | 57 | S-colon | Ⅲ | 2.5 | 110 | WT | n.d. | – | n.d. | – |
|  |  |  |  |  |  |  |  | 10.9 |  | 4.5 |  |
| 22 | M | 75 | Rectum | Ⅲ | 80.4 | < 20 | WT | n.d. | – | n.d. | – |
|  |  |  |  |  |  |  |  | 1 |  | 1 |  |
| 23 | M | 60 | S-colon | Ⅲ | 46.4 | < 20 | WT | n.d. | – | n.d. | – |
|  |  |  |  |  |  |  |  | 1.6 |  | n.d. |  |
| 24 | M | 58 | Rectum | Ⅲ | 55.8 | 67 | WT | n.d. | – | n.d. | – |
|  |  |  |  |  |  |  |  | 5.3 |  | 3.8 |  |
| 25 | M | 46 | Rectum | Ⅲ | 4.5 | 54 | WT | n.d. | – | n.d. | – |
|  |  |  |  |  |  |  |  | 4.3 |  | 4.3 |  |
| 26 | M | 71 | A-colon | Ⅳ | 5 | 60 | WT | n.d. | – | n.d. | – |
|  |  |  |  |  |  |  |  | 3.4 |  | 2.5 |  |
| 27 | M | 59 | Rectum | Ⅳ | 775 | 169 | WT | n.d. | – | n.d. | – |
|  |  |  |  |  |  |  |  | 19 |  | 6.5 |  |
| 28 | M | 64 | S-colon | Ⅳ | 7.8 | < 20 | WT | n.d. | – | n.d. | – |
|  |  |  |  |  |  |  |  | 1.6 |  | 4.2 |  |
| 29 | M | 72 | S-colon | Ⅳ | 183.8 | 1500 | WT | n.d. | – | n.d. | – |
|  |  |  |  |  |  |  |  | 263 |  | 43.6 |  |
| 30 | F | 55 | S-colon | Ⅳ | 1.7 | < 20 | WT | n.d. | – | n.d. | – |
|  |  |  |  |  |  |  |  | 3.1 |  | n.d. |  |
| 31 | M | 54 | S-colon | Ⅳ | 54.3 | 105 | WT | n.d. | – | n.d. | – |
|  |  |  |  |  |  |  |  | 12.2 |  | 4.3 |  |
| 32 | M | 61 | S-colon | Ⅳ | 2.4 | 2170 | WT | n.d. | – | n.d. | – |
|  |  |  |  |  |  |  |  | 419 |  | 15.7 |  |
| 33 | M | 39 | S-colon | Ⅳ | 647.8 | 1330 | WT | n.d. | – | n.d. | – |
|  |  |  |  |  |  |  |  | 194 |  | 5.6 |  |
| 34 | F | 69 | T-colon | Ⅳ | 75.3 | 359 | WT | n.d. | – | n.d. | – |
|  |  |  |  |  |  |  |  | 40.5 |  | 7.2 |  |
| 35 | M | 59 | S-colon | Ⅳ | 7317.9 | 1230 | WT | n.d. | – | n.d. | – |
|  |  |  |  |  |  |  |  | 80 |  | 7.4 |  |
| 36 | M | 69 | S-colon | Ⅳ | 4.1 | 194 | WT | n.d. | – | n.d. | – |
|  |  |  |  |  |  |  |  | 21.1 |  | 4.8 |  |
| 37 | M | 56 | T-colon | Ⅳ | 3690.1 | 476 | WT | n.d. | – | n.d. | – |
|  |  |  |  |  |  |  |  | 48.9 |  | 10.2 |  |
| 38 | F | 64 | A-colon | Ⅳ | 616.7 | 634 | WT | n.d. | – | n.d. | – |
|  |  |  |  |  |  |  |  | 93 |  | 12.3 |  |
| 39 | M | 61 | Rectum | Ⅳ | 60.7 | 454 | WT | n.d. | – | n.d. | – |
|  |  |  |  |  |  |  |  | 27.5 |  | 18 |  |
| 40 | F | 75 | A-colon | Ⅳ | 2950 | 1630 | WT | n.d. | – | n.d. | – |

Abbreviations used in Table S2: A-colon, ascending colon; S-colon, sigmoid colon; D-colon, descending colon; T-colon, transverse colon; CEA, carcinoembryonic antigen; WT, wild type; n.d., not detected; FA, fractional abundance.

**SUPPLEMENTARY FIGURES**


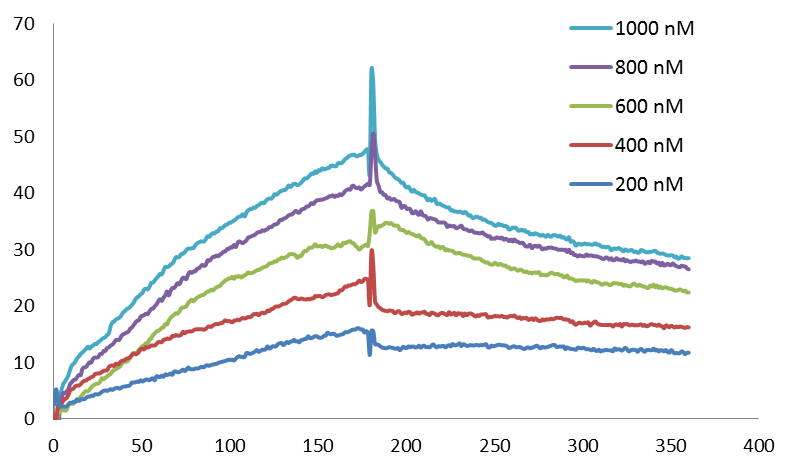

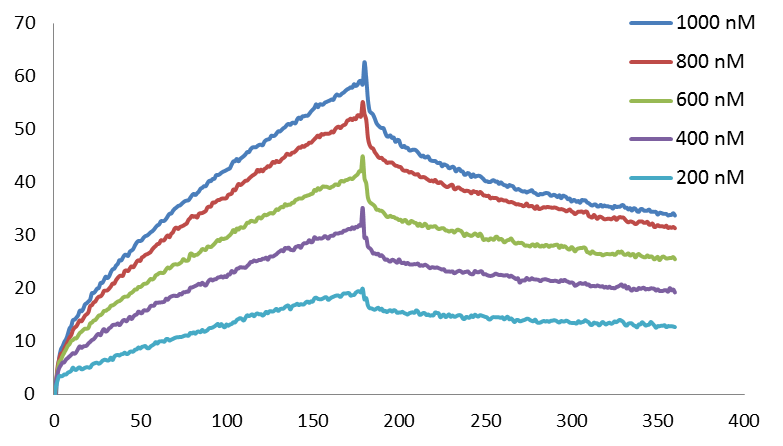

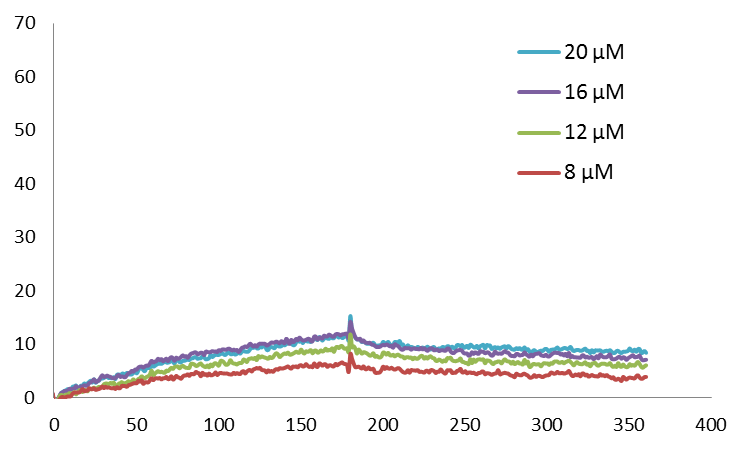


Time (s)

Time (s)

Time (s)

(a)

(b)

(c)

Response (RU)

Response (RU)

Response (RU)

**Figure S1**

Surface plasmon resonance sensorgrams for the interaction between KRAS 5 and hairpin DNAs containing the sequence (a) *KRAS* GAT mutation, (b) *KRAS* GTT mutation, and (c) *KRAS* GGT wild type immobilized on the surface of a sensor chip SA.　In (a) and (b), each of the five curves of the lowest, mid–low, middle, mid–high, and highest indicates concentrations of 200, 400, 600, 800, and 1000 nM PI polyamides, respectively. In (c), each of the five curves, the lowest, mid–low, mid–high, and highest indicate concentrations of 8, 12, 16 and 20 μM PI polyamides, respectively.

**Figure S2** Relationship between fold of enrichment assay and fraction of mutant alleles (duplex analysis).

(A) Fold enrichment of KRAS5 for G12V alleles. (B) Fold enrichment of KRAS5 for G12D alleles.

**Figure S3-1** One-dimensional plots of digital PCR analysis before and after enrichment assay.

(A, FAM-positive) Blue dots represent droplets containing *KRAS* mutation. (B, HEX-positive) Green dots represent droplets containing wild type *KRAS*.

**Figure S3-2** One-dimensional plots of digital PCR analysis before and after enrichment assay.

(A, FAM-positive) Blue dots represent droplets containing *KRAS* mutation. (B, HEX-positive) Green dots represent droplets containing wild type *KRAS*.
